# Temperature-induced configuration changes in hydrogel-coated coils and their relevance in embolization procedures

**DOI:** 10.1186/s42155-020-00189-0

**Published:** 2020-12-10

**Authors:** Ruben Lopez Benitez, Tomas Reyes del Castillo, Levent Kara, Joachim Kettenbach, Justus Roos

**Affiliations:** 1 Department of Radiology and Nuclear Medicine, Luzerner Kantonsspital, 6000 Luzern, Switzerland; 2 Stadtspital Triemli Zürich, Institute of Radiology and Nuclear Medicine, 8063 Zurich, Switzerland; 3 Zentralröntgeninstitut für Diagnostik, Interventionelle Radiologie und Nuklearmedizin, Landesklinikum Wiener Neustadt, 2700 Wiener Neustadt, Austria

**Keywords:** Interventional radiology, Embolization procedures, Hydro-coated coils, Temperature-induced configuration, Water immersion, Coil optimization

## Abstract

**Background:**

The present study attempted to demonstrate how the configuration of hydrogel-coated coils is influenced by different temperature exposures.

Thirty detachable hydrogel-coated coils were evaluated in an in vitro water immersion test under five different temperature ranges (22.6 °C, 37 °C, 40–50 °C, 50–60 °C, and 60–70 °C). The configuration changes were classified (configuration I, configuration II, and configuration III) according to the curling that occurred during 30 min of immersion. Configuration stability of five Hydrogel-coated coils was also evaluated in a two-step temperature immersion test.

**Results:**

All hydrogel-coated coils showed some configuration changes during water immersion. However, a logarithmic transformation of the time and temperature data showed a significant (*p* < 0.05) negative linear correlation between time and temperature for all coil configurations (configuration I: *R* = 0.97, configuration II: *R* = 0.98, configuration III: *R* = 0.97). The time needed to reach configuration III (complete coiling) was 160.4 ± 41.9 s at 37.5 °C (range: 100–205 s), 45.7 ± 22.2 s at 47.5 °C (range: 23–70 s), 20.2 ± 7.2 s at 57.5 °C (range: 14–32 s), and 10.3 ± 2.4 s at 67.5 °C (range: 7–13 s).

**Conclusions:**

Temperatures above 55 °C induced immediate configurational changes in the hydro-coated coils, achieving complete curling within less than 30 s. Temperatures near 36 °C (normal body temperature) require more time to reach optimal coil curling (configuration III). The optimization of HydroCoil preparation can reduce interventional procedural time and improve clinical results.

## Background

Vessel embolization using a coil technique is a challenging procedure that requires technical skills and a detailed knowledge of the properties of the materials employed. In order to achieve complete embolization of the target vessel and avoid complications, embolization materials should be selected according to their special features, taking into account either the clinical indication and/or the vascular bed anatomy (Orron et al., 2018).

Coils are one of the most frequently used materials for vascular occlusion. On a basic level, coils are metal filaments made of platinum, cobalt-chrome, or nitinol (Schelhorn et al., 2014; Pech et al., 2009). Recently, HydroCoils® (MicroVention Inc., Tustin, CA) have been introduced as a coil system designed to improve coil-packing density. This is achieved by a layer of hydrogel acrylic polymer surrounding a platinum core, which increases in thickness and diameter when in contact with liquid or blood (Fohlen et al., 2018).

An efficient HydroCoil configuration improves the embolization effect. An optimized configuration can save expensive materials and unnecessary coiling of the extended vascular segments (Lopez-Benitez et al., 2013). In the present in-vitro study, we attempted to precisely define how HydroCoil configuration can be influenced by exposure to different water temperatures.

## Materials

**Fig. 1:**
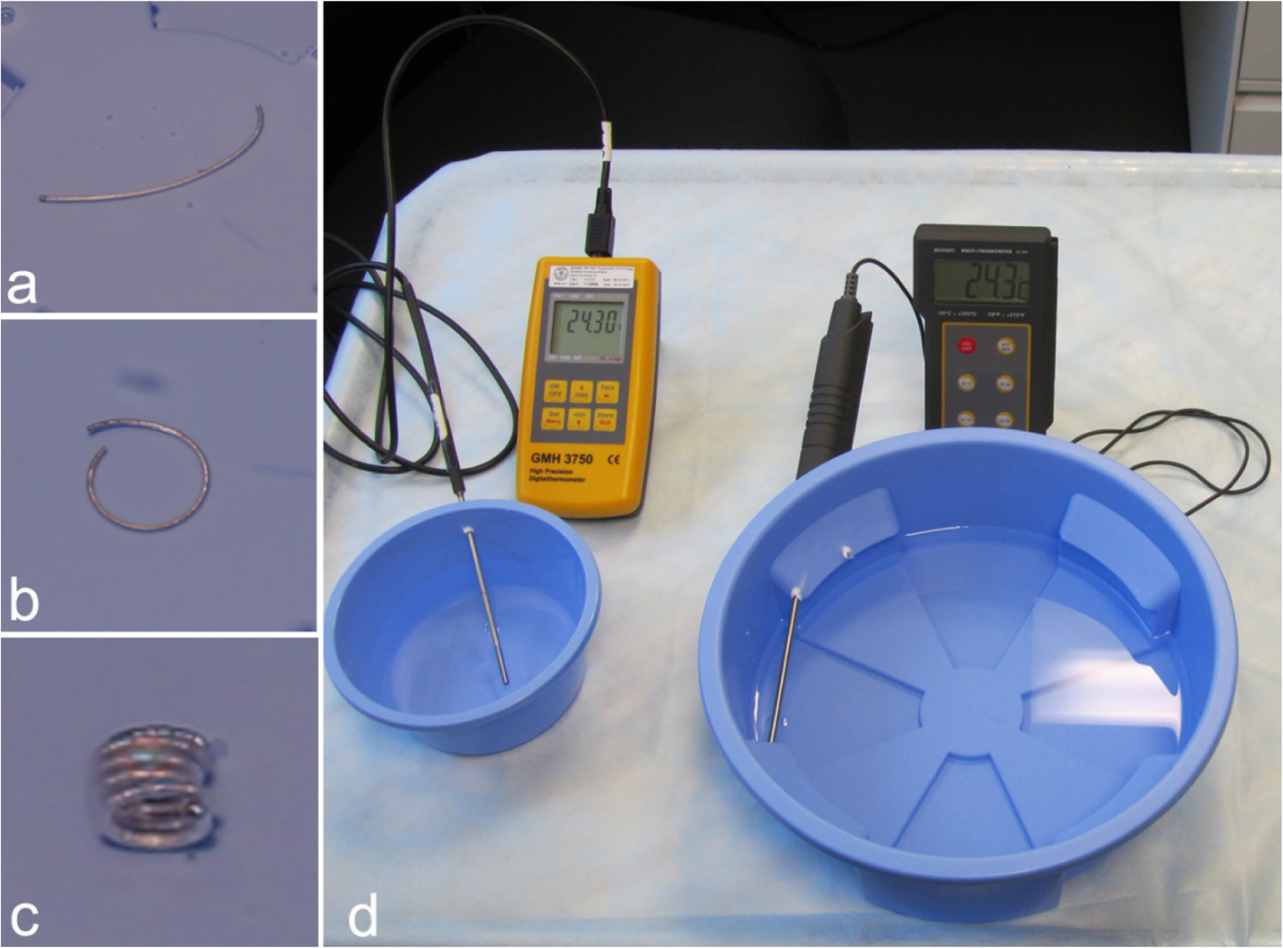
Classification of HydroCoil configurational changes: Configuration I, minimal curvature of the HydroCoil without reaching a first loop (**a**); Configuration II, first complete loop without complete curling of the HydroCoil (**b**); Configuration III, complete HydroCoil curling (**c**). **d** shows two different experimental open-fluid immersion chambers with their respective digital thermometer systems (the external thermostat and chronometers are not shown)

Thirty detachable HydroCoils were used in the present study. All were tested in an open-fluid thermostatic system consisting of the following parts: a polyurethane immersion chamber, a thermostatic system with a maximal temperature variation range of 0.5 °C (Voltkraft^GmbH^, Hirschau, Germany), and a digital thermometer with a measurement range of − 199.9 °C to + 850.0 °C (GMH 3750, Greisinger Electronic^GmbH^, Regenstauf, Germany) calibrated according to the Swiss certification (Cert. 112,954; Fig. 1).

The immersion chamber was filled with 500 ml of sterile 0.9% NaCl solution with 2000 units of heparin (Liquemin, Drossapharm^AG^, Basel, CH), simulating the exact conditions of the fluids used in conventional angiographic procedures.

Water temperature was regulated and maintained using an external thermostat (Voltkraft^GmbH^, Hirschau, Germany), thus ensuring qualitative and reproducible conditions. All measurements were recorded for subsequent analysis using a high-definition camera (Sony-HDV, Sony, Japan) and recorded on a DN-300a 250GB data video unit (Datavideo®, Utrecht, Holland). The timing of configuration changes was measured using a digital chronometer (T3-precision, Suunto™, Vantaa, Finland).

### Methodology

The experimental design consisted of two parts: a one-step temperature immersion test and a two-step temperature immersion test.

### One-step temperature immersion test

The time measurements were chronometrically registered from the time at which the hydrogel-coated coils were manually immersed into the fluid chamber (at the selected temperature range) until the time at which one of the configurations was achieved and no further changes were observed. In accordance with the study design, we performed the experiments under five different temperature ranges: 22.6 °C (room temperature), 37 °C (body temperature), 40–50 °C, 50–60 °C, and 60–70 °C.

Twenty-five (*n* = 25) HydroCoils (0.035″ wire diameter, 8-mm loop, 60-mm length) were analyzed with five in each temperature group. Each configuration change was classified as one of three morphologies (Fig. 1):

- Configuration I: Minimal curvature of the hydro-coated coil without reaching a first loop.
- Configuration II: First complete loop without complete curling of the hydro-coated coil.
- Configuration III: Complete/final hydro-coated coil curling.

Time measurements were stopped under one of the following conditions: 1) when the investigators (two observers: RLB and TR) confirmed that a complete and stable configuration of the coil (Configuration III) had been achieved or 2) at 30 min in cases of incomplete coil configuration. Values were measured and registered in minutes, seconds, and milliseconds.

### Two-step temperature immersion test

In the second part of the study, five (*n* = 5) detachable hydrogel-coated coils were analyzed to assess the configuration memory of the material. The detachable hydro-coated coils were first immersed in a 70 °C water bath. Once a completely coiled shape was reached (Configuration III), the coil was retracted into its plastic sheath and pulled out of the immersion chamber. After 10 seconds at room temperature (approximately 26 °C), the hydrogel-coated coil was immersed in a 37 °C bath and released from its sheath. The time period until the HydroCoil reached complete coiling was measured. The purpose of this test was to assess whether a HydroCoil that was completely configured once would be able to instantly regain that configuration after being released from its sheath at physiologic temperatures, simulating a clinical environment (i.e., the preparation of the HydroCoil outside of the patient and subsequent placement in the target vessel).

### Statistical analysis

All data were analyzed and evaluated using Stata software version 12.1 (Stata Corp. LP, College Station, TX, USA). The data values are expressed as means ± standard deviations.

## Results

**Fig. 2:**
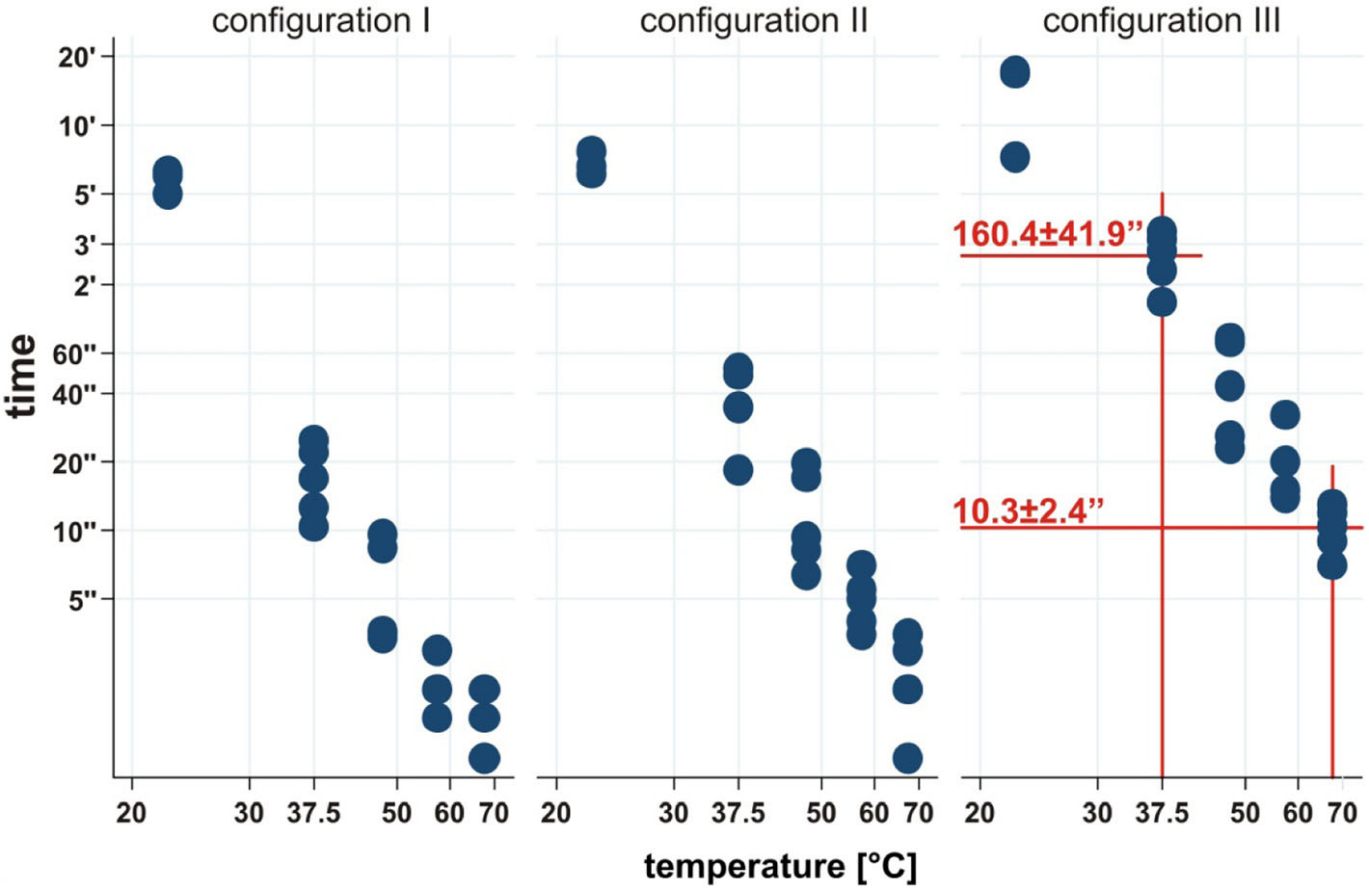
Temperature-induced HydroCoil configuration changes. Scatter plots of time needed to reach a specific coil configuration as a function of temperature. Note the use of a double logarithmic scale. The time points at which Configuration III was achieved at 37 °C and 70 °C are highlighted in red

**Fig. 3:**
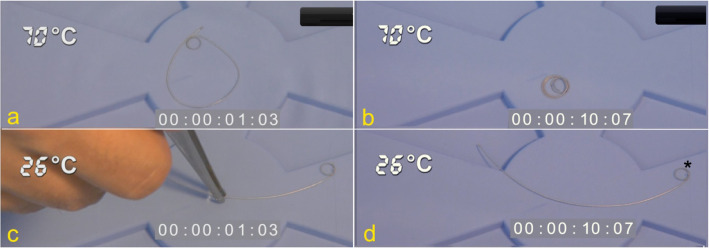
HydroCoil configuration changes according to different water temperatures. **a** shows a first loop configuration (Configuration II) after only 01:03 s in a 70 °C water immersion. **b** shows complete coiling (Configuration III) after only 10:07 s in a 70 °C water immersion. **c** shows a HydroCoil during the first second after immersion at 26 °C; only a discrete strain (Configuration I) was observed after 10:07 s in a 26 °C water bath immersion (**d**); this HydroCoil remained without configurational changes even after several minutes (not shown). *NOTE: All HydroCoils are designed with a preformed first loop at the tip of the filament (black asterisk); in normal conditions, this first loop facilitates the initial binding of the HydroCoil. This first loop should not be misinterpreted as a temperature-induced coil binding, which actually comprises the totality of the HydroCoil filament*

The measurements of time until certain coil configurations were reached in relation to the solution temperature are summarized in Table 1 and Fig. 2 (note the use of double logarithmic scales). In all temperature categories, all the HydroCoils showed some form of configuration change after immersion in water (Fig. 3). All five HydroCoils reached Configuration I at room temperature, whereas only three HydroCoils reached Configurations II and III. The average time needed to reach a particular configuration was 327 ± 38 s for Configuration I (range: 300–376 s), 408 ± 49 s for Configuration II (range: 367–462 s), and 820 ± 336 s for Configuration III (range: 433–1026 s). At temperatures above 37 °C, all HydroCoils reached Configuration III within 30 min. The individual times needed to reach Configuration III (complete coiling) were 160.4 ± 41.9 s at 37.5 °C (range: 100–205 s), 45.7 ± 22.2 s at 47.5 °C (range: 23–70 s), 20.2 ± 7.2 s at 57.5 °C (range: 14–32 s), and 10.3 ± 2.4 s at 67.5 °C (range: 7–13 s).

**Table 1:** HydroCoil configuration according to temperature increments

Temperature	Configuration I Average [sec] (data range)	Configuration II Average [sec] (data range)	Configuration III Average [sec] (data range)
22.5 °C	327 (300–376)	408 (367–462)	820 (433–1026)
37.5 °C	17.3 (10.4–24.8)	37.5 (18.5–51.4)	160 (100–205)
47.5 °C	5.7 (3.4–9.6)	12.2 (6.4–19.8)	45.8 (22.9–70.0)
57.5 °C	2.3 (1.5–3.0)	5.0 (3.5–7.0)	20.2 (14.0–32.0)
67.5 °C	1.5 (1.0–2.0)	2.3 (1.0–3.5)	10.3 (7.0–13.0)

Measurements of time until a certain coil configuration was reached in relation to one of five different temperature groups. Data are summarized in averages and ranges

As Fig. 2 shows, there was a strong relationship between the temperature of the water bath and the time needed to reach a certain coil configuration. After a log transformation of time and temperature, the data showed a significant (*p* < 0.05) negative linear correlation between time and temperature for all coil configurations (Configuration I: *R* = 0.97, Configuration II: *R* = 0.98, Configuration III: *R* = 0.97).

For the two-step temperature immersion test, the temperature of the water bath was intentionally higher (above 70 °C) in order to achieve Configuration III in a short period of time (4.1 ± 0.9 s; range: 3.2–5.4 s). After the HydroCoil had retracted into its sheath and remained at room temperature (26 °C) for 10 seconds, it was reimmersed in the 37.5 °C water bath. All the examined HydroCoils immediately regained the fully coiled configuration when reimmersed in the 37 °C bath. Therefore, no time measurements were possible.

**Fig. 4:**
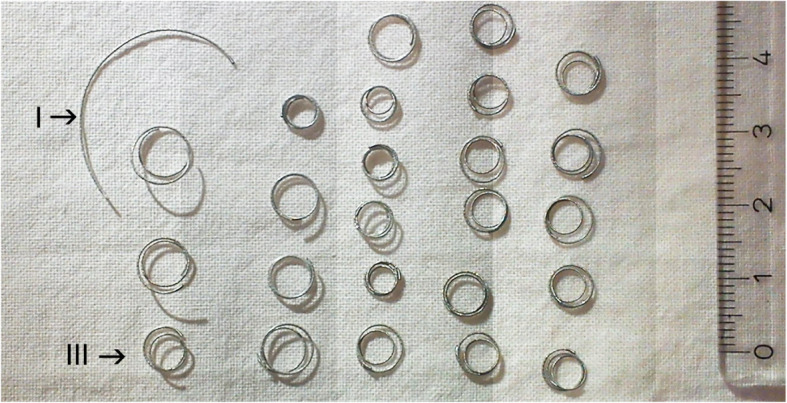
Differently configured HydroCoils (Configurations I and III) three months after the experimental assay. In all the cases, the morphology obtained during the experiment remained unmodified. Configuration II is not illustrated

All the HydroCoils were kept in a case at room temperature without humidity exposure and macroscopically analyzed 3 months after the experimental assay. There were no reoccurrences of configurational shape changes. The HydroCoils that had acquired Configurations I, II, and III after the experiment retained the same configurations (Fig. 4).

## Discussion

In the present study, we evaluated how a HydroCoil configuration is influenced when immersed in a water bath at different temperatures.

The timing of HydroCoil configuration is influenced by the temperature of a sterile water bath outside the patient, and the coil materials have been verified to have a memory effect. This enables one to apply these materials more efficiently (i.e., through coil packing and the use of less material) and to achieve a more targeted embolization, thus avoiding the embolization of extended vascular segments.

There are two clinical situations in which the modification of temperature applied to a HydroCoil can influence embolization performance. The first example is the use of detachable HydroCoils in the setting of protective embolization in selective internal radioembolization therapy (SIRT) procedures (Lopez-Benitez et al., 2013). In this case, the preparation of the HydroCoil by brief immersion in a sterile water bath at 55–70 °C allows for a fast preconfiguration and compact design of the HydroCoil, which allow for very accurate deployment in the proximal part of the gastroduodenal artery (GDA; Fig. 4).

**Fig. 5:**
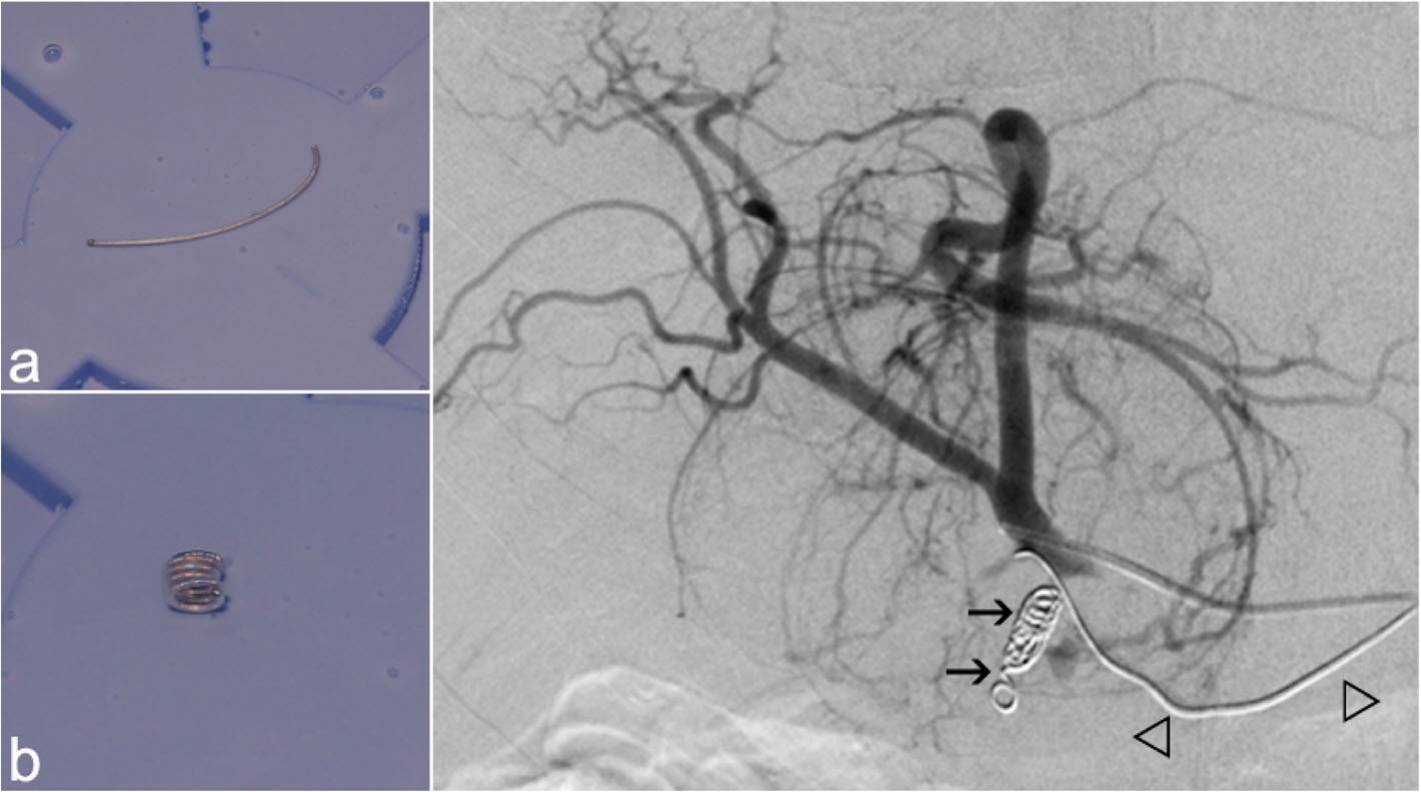
Correlation of two different HydroCoil configurations in a patient prepared for a selective internal radioembolization procedure. The filament-like HydroCoil configuration (**a**) allows access to narrow vessels, such as the right gastric artery, in a retrograde fashion (arrowheads) using the expandable capabilities of the HydroCoil to induce vascular occlusion. After preparation at 55–70 °C (**b**), the compact configuration of a curled HydroCoil is useful for the proximal occlusion of the gastroduodenal artery (arrows) to avoid particle reflux during the radioembolization procedure

Another example is the embolization of narrow vessels that are not accessible even with a microcatheter. In such cases, other embolic materials (e.g., particles or glues) usually increase the risk of complications, such as non-target embolization or tissue necrosis (Abdalkader et al., 2020; Kim et al., 2017; Lopez-Benitez et al., 2007). In our department, these narrow vessels are successfully embolized using a non-configured detachable HydroCoil, taking advantage of the filament-like configuration to navigate through the vessel; the occlusion results from the secondary intravascular expansion of the hydrogel coating. Even if the desired embolization of a vascular segment is located several centimeters away from the tip of the microcatheter, embolization is possible because the HydroCoil is able to navigate as a guide wire through the vascular bed until the desired vessel segment is reached. In this clinical setting, an early configuration of the HydroCoil must be avoided to allow sufficient time to place the coil. Therefore, the preparation of the HydroCoil by immersion in a sterile water bath at room temperature (≈26 °C) will not significantly induce changes in its configuration; this makes it possible to push the HydroCoil as a guide wire into distal segments (Fig. 5).

Interestingly, when a patient is embolized in a real clinical situation, the elapsed time during the entire passage of the HydroCoil through the microcatheter to the desired embolization area could theoretically induce recooling in the previously prepared HydroCoil that had been immersed in a warm bath (> 55 °C). It is conceivable that this change in temperature (from > 55 °C to 37 °C, body temperature) should result in a loss of the previously induced curling capability.

The results of the two-step immersion test revealed that the HydroCoils had excellent thermic memory, as all of them recovered their complete configurations after a cooling cycle of 10 seconds at room temperature (≈26 °C) and reimmersion in a 37 °C water bath. After the experiments were finished, all the HydroCoils were kept for 3 months at room temperature. In all cases, the final configurations (Configurations 0 to III) obtained during the experiment did not change (Fig. 5). In other words, once a HydroCoil is preheated and has taken on a curled configuration, it will not return to a straight form even if it is cooled down to room temperature.

In many institutions, the use of steam is common when the induction of configurational changes in materials is required (e.g., coil preshaping and catheter shaping). For the purposes of this study, steam was not used to increase the temperatures of the HydroCoils mainly because it is not possible to maintain a controlled, sustained baseline temperature under experimental conditions (this requires exact values). In liquid water, temperature can be regulated and maintained as needed for long periods of time with the use of a thermostat, ensuring qualitative and reproducible conditions.

In our institution, we perform more than 250 embolization procedures every year. HydroCoils have been used in many of these procedures, depending on the clinical indication and the anatomical conditions of each particular case. We have observed that the total number of coil devices needed for embolization and the time required for complete vascular occlusion have reduced significantly since we started modifying the temperature before HydroCoil deployment. Moreover, the acceptable handling of this material and the lower late recanalization rates offered by their expandable capabilities make HydroCoils optimally valuable embolization materials (Maleux et al., 2013; Brinjikji et al., 2015; Ferral, 2015).

## Conclusion

Temperatures above 55 °C induce immediate configuration changes in HydroCoils, achieving complete coiling in less than 30 s. Optimization during HydroCoil preparation can reduce total interventional procedure times. Moreover, knowledge of thermically induced configurational changes can enable the use of this material in different clinical settings.

## Data Availability

Not applicable.

## Abbreviations

SIRT: Selective internal radioembolization therapy
GDA: Gastroduodenal artery
